# Somatosensory Evoked Potentials suppression due to remifentanil during spinal operations; a prospective clinical study

**DOI:** 10.1186/1748-7161-5-8

**Published:** 2010-05-12

**Authors:** Irene Asouhidou, Vasilios Katsaridis, Georgios Vaidis, Polimnia Ioannou, Panagiotis Givissis, Anastasios Christodoulou, Georgios Georgiadis

**Affiliations:** 1 2nd Department of Anesthesiology "G.Papanikolaou" General Hospital, Exohi Thessaloniki, Greece; 2 Department of Neurosurgery "G.Papanikolaou" General Hospital, Exohi Thessaloniki, Greece; 3 1st Orthopaedic Department, Aristotle University of Thessaloniki G Papanikolaou Hospital, Exohi, Thessaloniki

## Abstract

**Background:**

Somatosensory evoked potentials (SSEP) are being used for the investigation and monitoring of the integrity of neural pathways during surgical procedures. Intraoperative neurophysiologic monitoring is affected by the type of anesthetic agents. Remifentanil is supposed to produce minimal or no changes in SSEP amplitude and latency. This study aims to investigate whether high doses of remifentanil influence the SSEP during spinal surgery under total intravenous anesthesia.

**Methods:**

Ten patients underwent spinal surgery. Anesthesia was induced with propofol (2 mg/Kg), fentanyl (2 mcg/Kg) and a single dose of cis-atracurium (0.15 mg/Kg), followed by infusion of 0.8 mcg/kg/min of remifentanil and propofol (30-50 mcg/kg/min). The depth of anesthesia was monitored by Bispectral Index (BIS) and an adequate level (40-50) of anesthesia was maintained. Somatosensory evoked potentials (SSEPs) were recorded intraoperatively from the tibial nerve (P37) 15 min before initiation of remifentanil infusion. Data were analysed over that period.

**Results:**

Remifentanil induced prolongation of the tibial SSEP latency which however was not significant (p > 0.05). The suppression of the amplitude was significant (p < 0.001), varying from 20-80% with this decrease being time related.

**Conclusion:**

Remifentanil in high doses induces significant changes in SSEP components that should be taken under consideration during intraoperative neuromonitoring.

## Introduction

Electrophysiological monitoring is applied during spinal surgery in order to assess the nervous tissue at risk for injury in a patient who is unable to respond due to anesthesia. There are several tests that can be performed intraoperatively to indicate a probable spinal injury; the so-called "wake up" test is time consuming and can not be performed at any time or in the emergency setting while motor evoked potentials (MEPs) are extremely sensitive to anesthetic agents. Somatosensory evoked potentials (SSEP) measure the integrity of the sensory pathways in the dorsal columns of the spinal cord, by stimulating a peripheral sensory nerve and measuring the electrical response in the brain. The introduction of SSEP monitoring to spinal surgery has significantly reduced the rate of intraoperative injury. A survey of the Scoliosis Research Society and the European Spinal Deformities Society documented a reduction in injury rate from 0.7-4.0% in the pre-SSEP monitoring days to less than 0.55% with SSEP monitoring [1].

SSEP are less affected by anesthetic agents than MEP [2]. The depressant effect of volatile anesthetics on evoked potentials is well known [3-5]. Recent studies consider total intravenous anesthesia (TIVA) with the combination of propofol and fentanyl as more appropriate for intraoperative neuromonitoring [4-10]. However, intravenous anesthetics affect SSEPs as well, in a dose-related fashion [5,7,8,10]. The effect of propofol on SSEPs latency and amplitude has been already addressed. Propofol produces from minimal to less than 10% suppression of SSEP amplitude [1,5,7,8,10].

Opioids are commonly used to supplement anesthetic agents during neuromonitoring and are considered to have only minimal effects on anesthetic-sensitive potentials. Different doses of fentanyl have been noted to cause prolonged amplitude depression [11,12].

Remifentanil is a newer short-acting opioid and its pharmacokinetics and pharmacodynamic characteristics make it unique for use in TIVA. It may also provide an excellent adjunct in situations where muscle relaxation must be avoided and minimal dosing of anesthetic agents is required. Animal data suggest that remifentanil is less suppressive than other opioids on MEPs but does not affect the SSEP [13,14]. Recent studies in humans have shown some dose-dependent reduction on the motor system when remifentanil was used as a single agent but there is not any published clinical trial of the effect of remifentanil on SSEP during general anesthesia [15].

Patients undergoing spinal surgery usually experience severe pain and large doses of opioids are administered intraoperative in combination with other anesthetic agents. Therefore it is essential to justify in which way the SSEPs are affected by opioids so that clinicians can make more informed interpretations of SEP changes. This study was designed to evaluate the effect of remifentanil on SSEP's latency and amplitude during propofol anesthesia in patients undergoing spinal surgery.

## Materials and methods

In this prospective study, intraoperative data of 10 otherwise healthy patients (mean age 42.2 ± 30.7) years, range of 13-88 years (4 females and 6 males) were analyzed. The procedures performed included surgery on the thoracic/lumbar spine for scoliosis or stenosis with instrumentation.

Approval of the local ethics committee and written informed consent were obtained. Patients with ASA physical status > 2, Body Mass Index (BMI) over 30, indication for rapid sequence induction, recent administration of central nervous system affecting drugs, or neurological or psychiatric diseases were excluded from this investigation.

All patients received 0.1 mg/kg oral dose of diazepam as premedication approximately 2 hours before surgery. No other sedatives or centrally acting agents were given before induction of anesthesia. Perioperative monitoring included continuous electrocardiogram of five leads-ECG, heart rate (HR), noninvasive and invasive (arterial line) systolic, diastolic and mean arterial pressure (MAP), end-tidal CO_2_, pulse oximeter probe (SpO_2_) oesophageal temperature (Datex Ohmeda) and ourine output. Intraoperative normothermia was actively maintained with a forced air warming blanket. The depth of anesthesia was monitored with the bispectral index (BIS) (target 40 to 50) through 4 skin electrodes placed in a 2-channel referential montage on patients' foreheads. All leads were connected to an electroencephalographic monitor (Aspect Medical System Inc, GR, Version 3.2).

Cardiac output (CO) was monitored through pulse contour analysis, in order to ensure that CO is not significantly influenced by the high dose of remifentanil. A FloTracTM sensor kit (Edwards Lifesciences) was connected to the arterial line and connected to the VigileoTM monitor programmed with the 3.02 version (2009) of the software for this device. Patient data (age, gender, body weight, and height) were entered and after checking the arterial line waveform fidelity, the system was zeroed and cardiac output measurement initiated.

General anesthesia was induced with propofol (2 mg/Kg), fentanyl (2 mcg/Kg) and cis-atracurium (0.15 mg/Kg). After anesthesia induction the trachea was intubated, and mechanical ventilation with intermittent positive pressure ventilation was started at a tidal volume of 7 mL/kg of IBW and with respiratory frequency adjusted to maintain end-tidal CO_2 _between 30 and 35 mmHg in a semi closed circuit with 2.0 L/min of fresh gas flow. The lungs were ventilated with air/O_2 _mixture and ventilation was adjusted to an EtCO_2 _tension of 30-35 mmHg, in order to achieve mild vasoconstriction that minimizes the blood loss and provides a better surgical field. Anesthesia was maintained with continuous intravenous infusion of remifentanil and propofol (30-50 mcg/kg/min). Use of neuromuscular block was avoided after intubation. The dose of remifentanil (0.8 mcg/Kg/min) was chosen, based on our previous clinical experience of the dose (about 0.5-0.8 mcg/kg/min) that is usually required to control the intraoperative pain stimulus.

Due to the fact that false positive readings and perioperative SSEP changes are elicited from factors such as hypothermia, anemia, hypocapnia (PaCO_2 _< 30 mmHg) or hypotension, the temperature was maintained over 36°C, the transfusion threshold at Hct < 30 mg/dl, and the mean arterial pressure (MAP)≥60 mmHg with vasoactive drugs (esmolol and ephedrine).

### SSEP protocol

The evoked response is recorded as a plot of signal amplitude (in mcV) versus latency (in msec), which is the time elapsed from stimulus delivery to arrival of the impulse at the recording electrode. An increase in the latency of 10% and a decrease in amplitude of 50% are considered significant [16]. Such changes reflect loss of the integrity of the neural pathway and a reason for intervention by the surgical team. In case that changes of SSEP components were over that limits, surgeons recheck the position of instrumentation; if they were sure that these changes were not due to their manipulations but due to anesthetic agents the operation was continued.

Measurement of the SSEP was used to monitor the function of the spinal cord throughout the operation. Subdermal stainless steel needle electrodes were percutaneously placed for stimulating the peripheral nerves and recording from the somatosensory cortical sites. Stimulation of the posterior tibial nerve (P37) with supramaximal constant-voltage single-pulse stimuli of 0.2 ms, at 20 per second, was applied alternately to both legs. For intraoperative assessment, a total of 300 intraoperative SSEP measurements from 10 patients were analyzed; a mean average of 12 +/- 4 of each tibial nerve was performed per operation. The SSEP amplitude was measured as the difference in microvolt (mcV) between the peak and trough deflections. The latency was measured as the time elapsed between stimulation and the first peak (msec). The changes in the minimum stimulus intensity (that is, threshold level) required to evoke elicit amplitude were also evaluated. The recordings were stored on the computer and were analyzed as a batch by an investigator blinded to the treatment condition. All markers were set by a single person to reduce variability.

Right after intubation continuous intravenous infusion (civ) of propofol was initiated in order to maintain a BIS value between 40-50. The patients were positioned on the table in spine-prone position and the first measurement of SSEP was done at that time; this first measurement was considered the baseline SSEP (SSEP_b_). The first SSEP measurement (SSEP_b_) was performed 30 minutes after induction in anesthesia. This time is enough to wash out the anesthetic agents administered to perform induction in anesthesia. After the baseline measurement civ of remifentanil in dose of 0.8 mcg/kg was initiated. As remifentanil plasma concentration reaches a steady state within 10 minutes of infusion, the second measurement was taken 15 minutes after initiation of civ of remifentanil (SSEP_r_).

### Statistical analysis

Data were expressed as mean ± SD (in all cases n = number of patients) and were analyzed statistically using the SPSS program package, version 16.0. Differences in categorical data were evaluated using the student t test. A beta error level of 15% or statistical power of 85% and an a-level of 0.05 was used to calculate the sample size of this study. The p-level was set at 0.05.

## Results

Ten patients were included in the study and all of them completed the study. Their demographic characteristics are shown in Table 1. Satisfactory SSEPs were recorded in all patients, allowing adequate monitoring at all times. Despite the high doses of remifentanil that were used in this study, we did not notice any episodes of bradycardia or low cardiac output even though the mean arterial pressure presented statistically significant difference (p = 0.005) (Table 2). All procedures were carried out without any surgical or anesthesiologic complications.

**Table 1:** Demographic data of patients

n	10
Age/range mean ± SD	13-88 42.125 ± 30.7

M:F	4:6

ASA I	7

ASA II	3

Weigh (Kg)	58.5 ± 13.07

BMI (kg/m^2^)	23.5 ± 3.55

High (cm)	157.375 ± 8.61

Duration of anesthesia (minutes)	237.5 ± 63.22

Duration of operation (minutes)	171.5 ± 43.19

Type of surgery	
Scoliosis thoracic	3
Lumbar spinal fusion	7

BMI: Body Mass Index, M:F, Male:Female, n = number of patients.

**Table 2:** Haemodynamic data of patients receiving remifentanil 0.8 mcg/kg/min

N	HRb (beat/min)	HRr (beat/min)	COb (L.min^-1^)	COr (L.min^-1^)	MAP_b_ (mmHg)	MAP_r_ (mmHg)
1	51	46	3.05	2.55	82	67

2	65	56	4.15	3.9	71	60

3	53	50	4.6	4.25	102	82

4	51	50	2.7	2.75	66	66

5	78	56	4.4	3.3	90	64

6	69	55	4.7	4.0	69	45

7	72	58	5.7	3.9	79	72

8	67	58	4.1	3.1	83	70

9	72	61	3.4	3.0	91	74

10	68	57	3.6	2.9	**86**	68

mean ± SD	64.6 ± 9.6	54.7 ± 4.59	4.17 ± 0.947	3.47 ± 0.631	81.9 ± 11.1	66.8 ± 9.73

Co_b_: Baseline Cardiac Output, Co_r_: Remifentanil group Cardiac Output, HR_b_: Heart Rate baseline, HRr: Heart Rate remifentanil group, MAP_b_: Mean Arterial Pressure baseline, MAPr: Mean Arterial Pressure remifentanil group, n = number of patients.

**Figure 1:**
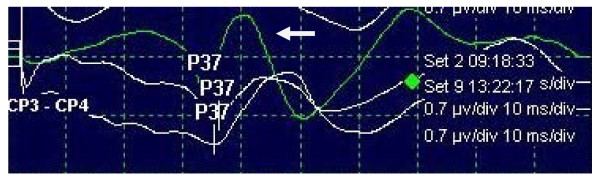
**Image of amplitude decrease after 244 minutes civ remifentanil (Set 9)**. White arrow indicates the baseline wave (Set 2). The prolongation of latency is also demonstrated.

Continuous intravenous infusion of remifentanil induced significant suppression of SSEP amplitude (p = 0.001). The waves of P37 amplitude were strongly reduced in comparison to baseline waves with administration of remifentanil. The decrease of amplitude was between 20%-80% (mean 50%). In one patient the amplitude diminished to zero for a period of about 10 minutes, not due to any surgical complication (Figure 1, patient 5). Fifteen minutes later the value of the amplitude increased to 0.2 mcV, without any alteration in remifentanil dose and remained at this value till the end of the operation. However this last value of amplitude represented a reduction of 65% from the baseline value.

**Figure 2:**
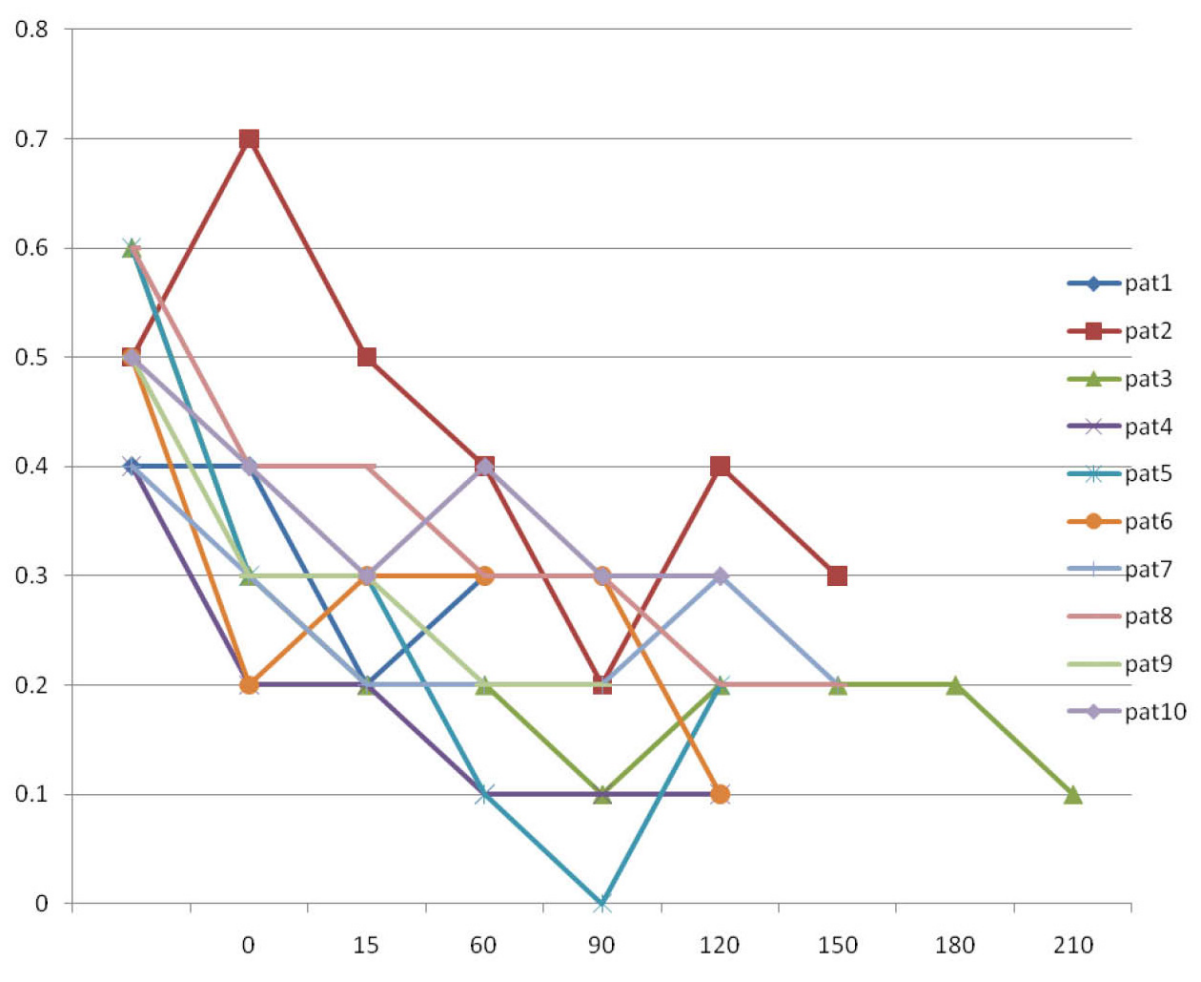
**Graphical plot of the amplitudes (axis y, μV) relative to the duration of remifentanyl infusion (axis x, sec)**.

Intraoperative tibial SSEP latencies were not significantly prolonged (p = 0.774) (Figure 2). The increase in latency was less than 10% which is the threshold to be considered as clinically significant with the prolongation being almost 1 msec. We also noted significant linear time related changes in P40 amplitudes (figure 2).

The effect of remifentanil on SSEP components in all patients is illustrated in Figure 2.

## Discussion

Neuromonitoring in the operating room allows for on-line surveillance and early diagnosis of spinal cord dysfunction and aims to provide warning signals before an irreversible damage has occurred. SSEP monitoring has been shown to be feasible during administration of inhaled or intravenous agents [3-5,11,12]. Since the use of volatile anesthetics is not recommended when neuromonitoring is performing, it is essential to titrate the doses of remifentanil during propofol anesthesia to levels that do not significantly affect the SSEP.

All lipophilic agents interfering with neuron membranes also interfere with subcortical conduction and therefore cause an increase in latencies [17]. According to some authors the application of low doses of fentanyl is not supposed to induce the predictable changes on SSEP latencies [10,18,19]. Strahm et al found that fentanyl produces significant changes of SSEP latency but less than 3%. The mean changes of all the amplitudes were less than 10% compared to the preoperative values but fentanyl was administered in different doses in every patient [20]. In a study comparing amplitudes between patients receiving either fentanyl or remifentanil, during spine surgery, it was found that amplitudes were better preserved in the remifentanil group [21]. However this study did not provide any details about the total dose of opioids administrated intraoperatively. It has also been shown that high doses of fentanyl decrease the amplitude by 60-70% in patients receiving 74 mcg/kg fentanyl in induction [22].

Remifentanil is the less lipophilic opioid compared to fentanyl, sufentanil or alfentanil, and this makes it the ideal opioid component of TIVA anesthesia during neuromonitoring. Our data demonstrate that remifentanil in a high dose induces significant suppression in amplitude of SSEP; in some patients even over 50%; which is the threshold to be considered clinically significant [17]. Also remifentanil induced prolongation of latency which however did not exceed the limit of 10%. Since remifentanil produces changes in SSEP's components, especially in amplitude, remifentanil should be combined with other agents, such as esmolol, in order to titrate remifentanil to a smaller dose. Schmidt et al demonstrated that remifentanil administrated as a single agent in a dose of 0.65 mcg/kg increases the SSEP amplitude [23]. However, when remifentanil was administered at higher doses (1 mcg/kg/min), during isoflurane anesthesia, the amplitude was reduced [24]. A study in rats demonstrated that remifentanil did not affect the SEP amplitude or latency when administered alone or when it was co-administered with propofol [14]. In this study ketamine was used which increases the amplitude [25]. The latency was increased approximately 1 msec which is a small but clinically significant change according to Kalkman et al [22].

Our data also demonstrate that remifentanil has an influence on both peak amplitude and latency of SSEP. This fact is a useful intraoperative diagnostic tool; a latency increase without any proportional amplitude decrease of a given peak means a delay of activation, without destruction, of this peak generator. As far as cortical or brain-stem grey matter generators are concerned, this implies that the dysfunctional site lies somewhere in the white matter afferents of this structure (spinal cord, brain-stem, cerebral hemispheres). A typical example of such a situation is the cortical SEP alterations occurring after spinal cord compression or ischemia. By contrast, a pathology involving only a grey matter generator and not its white matter afferents can cause an amplitude decrease or the disappearance of the corresponding peak, but there is no reason for the latency of this peak to be considerably increased [17].

There are some limitations of this study that should be recognized. The population study was small and future studies should be scheduled with biger one. Also further measurements should be performed using lower dose of remifentanil in order to define the threshold of amplitude changes due to remifentanil. We did not use target controlled infusion (TCI) for calculating the dose of propofol and remifentanil because TCI resulted in higher propofol consumption and delayed recovery probably due to inaccurate prediction of propofol effect site concentration [26].

In summary our study demonstrates that remifentanil in quite high doses suppresses the amplitude of SSEP recorded from the posterior tibial nerve during spinal surgery. The clinical implication of this finding lies in the fact that these changes in some cases exceeded the limit of clinical significance. This effect should be taken under consideration in order to perform reliable neuromonitoring while avoiding false positive results.

## Competing interests

The authors declare that they have no competing interests.

## Authors' contributions

AI, KV and VG participated in the design of the study and drafted the manuscript. GP, IP, CA and GG have been involved in drafting the manuscript. All authors read and approved the final manuscript.
